# mums2: an R package for database-dependent and -independent analysis of untargeted metabolomics data

**DOI:** 10.1128/mra.00780-26

**Published:** 2026-10-14

**Authors:** Gregory Johnson, Allison R. Mason, Joseph M. Krampen, Jennifer N. T. Nguyen, Marcy J. Balunas, Patrick D. Schloss

**Affiliations:** 1 Department of Microbiology & Immunology, University of Michigan 1259, Ann Arbor, Michigan, USA; 2 Department of Medicinal Chemistry, University of Michigan 1259, Ann Arbor, Michigan, USA; Wellesley College, Wellesley, Massachusetts, USA

**Keywords:** metabolomics, microbiome, LC-MS/MS, untargeted, bioinformatics

## Abstract

mums2 is an open-source R package that enables users to analyze data from untargeted metabolomics analyses. mums2 provides features that parallel existing tools for analyzing 16S rRNA gene sequence data, including data curation, annotation, clustering of features into operational metabolomic units (OMUs), and analyses based on ecological metrics.

## ANNOUNCEMENT

Metabolomics, the analysis of the metabolites within biological samples, has become especially popular among microbiologists seeking to integrate the structure and function of microbiomes in diverse environments (e.g., 1–3). Similar to how next-generation sequencing has facilitated a rapid advance in sequence-based analysis of microbiomes, advances in liquid chromatography-tandem mass spectrometry (LC-MS/MS) have the potential to revolutionize metabolomic analyses. Unfortunately, some of the most widely used tools for analyzing LC-MS/MS data are proprietary and expensive or require uploading data to a third-party website (4, 5). Other software options lack the features needed to conduct ecological analyses of a large number of samples (6, 7). We created mums2 as an open-source R package, allowing researchers to analyze untargeted metabolomics data generated using LC-MS/MS in a manner that parallels how they analyze microbiome data within their local computing environment.

mums2 allows users to curate LC-MS/MS data, annotate features, cluster features into operational metabolomic units (OMUs) based on their distance to each other, and compare collections of samples based on annotations or OMU counts using popular ecological metrics (e.g., Shannon diversity index, Bray-Curtis distances). Data curation can be performed using mpactR, which filters features based on their abundance and reproducibility across technical replicates (8). Structural annotations are made using a dot-product-based method that calculates cosine scores (9). The package provides users access to the Human Metabolome Database (HMDB) (10) and MassBank (11) databases, but any similarly formatted database can be used. A *de novo* molecular formula prediction is made for each feature using a fragmentation tree-based algorithm (12). To generate OMUs, MS2 fragmentation patterns are aligned (13), and the distances between the patterns are calculated using either the cosine or maximum entropy scores (14). These distances are clustered into OMUs using the OptiClust algorithm (15), which is implemented in the clustur R package (16). By creating mums2 as an R package, users can leverage the expansive ecosystem that includes the popular vegan package for ecology analyses, statistical analysis tools, and packages for data visualization.

The mums2 website provides installation instructions and a detailed vignette on how to use the package’s functions (https://www.mums2.org/mums2/). The mums2 workflow requires two types of data. The first is a data frame with MS1 and MS2 peak data, which can be generated using vendor-specific software such as Progenesis QI (Waters) and MetaboScape (Bruker) or web-based software including msConvert (17) and MZMine (6). The second is a data frame with metadata describing the experimental and run conditions. Instructions on how to generate and format these files are available on the package’s website. An example workflow is provided in the package’s vignette that demonstrates the package’s features using a previously published data set analyzing the metabolome of *Botryllus schlosseri* (18).

mums2 can be used on any operating system with only R dependencies, which are installed with mums2. The package makes use of the Rcpp and RcppThread R packages, which accelerate the performance of the functions and allow parallelization of the computationally intensive steps. It also makes use of the mpactR and vegan packages to implement curation and analysis steps, respectively. In our experience, a data set with 267 samples on 14 threads required 7.6 min of runtime and 6 GB of RAM, with 5 min of that used to predict the molecular formulas. The package’s functions output data structures that can be directly used with tools from R’s tidyverse.

## Data Availability

mums2 can be accessed through the Comprehensive R Archive Network (CRAN; DOI: https://doi.org/10.32614/CRAN.package.mums2) and developmental versions are available through the project’s GitHub website (https://github.com/mums2/mums2). The package’s GitHub repository includes the package’s source code, the website’s source code, automated tests written using the testthat R package, and an issue tracker where users can post questions, bug reports, and suggestions for future features. A pkgdown version of the documentation is hosted at (https://mums2.org/mums2). The mums2 package is available under the GNU General Public License (v3).
